# Colony size and brood investment of *Myrmica rubra* ant colonies in habitats invaded by goldenrods

**DOI:** 10.1007/s00040-018-0612-0

**Published:** 2018-02-26

**Authors:** I. M. Grześ, P. Ślipiński, H. Babik, D. Moroń, B. Walter, G. Trigos Peral, I. Maak, M. Witek

**Affiliations:** 1 Department of Environmental Zoology, Institute of Animal Sciences, Agricultural University, Al. Mickiewicza 24/28, 30-059 Kraków, Poland; 2 Museum and Institute of Zoology, Polish Academy of Science, Wilcza 64, 00-679 Warsaw, Poland; 3 Institute of Systematics and Evolution of Animals, Polish Academy of Sciences, Sławkowska 17, 31-016 Kraków, Poland

**Keywords:** Colony growth, Life-history traits, Social insects, *Solidago*, Reproduction

## Abstract

Ant richness and abundance are negatively affected by the invasion of alien goldenrods (*Solidago* sp.). However, little is known about the mechanisms standing behind the impact of the invaders on ant life history, such as colony investments in growth and reproduction. We examined this problem of the investments of *Myrmica rubra* ant colonies living in different grasslands invaded and non-invaded by goldenrods. Altogether, 47 colonies were analysed; and for each colony, we calculated the number of queens, workers and the production of young workers, gynes, and males. We found that colonies from invaded meadows are smaller in size, but have a similar number of adult queens compared to colonies from non-invaded sites. We also found different brood investments among colonies from invaded and non-invaded meadows—colonies from non-invaded meadows produce more young workers and invest more in growth, whereas colonies from invaded meadows invest more in reproduction through higher gyne production. Male production was at a similar level in colonies from both habitat types. The observed patterns may be explained by the effect of various environmental factors occurring in both grassland types, such as stress in changed habitats, higher competition among gynes in non-invaded grasslands, or finally, by the adaptive colony-level response of ants to stress. The higher production of gynes observed in the invaded grasslands may support dispersal and enhance the probability of establishing a colony in a more favourable location.

## Introduction

By definition, invasive species are those that have the capacity to spread in non-native areas and they usually displace native species through competition, leading to ecological disturbances in the new environment. The invasion can cause both biotic and abiotic stresses that in most cases is not easily avoided by native species (reviewed in Litt et al. 2014).

Many ant species can become invasive when introduced to new areas (Holway et al. 2002; Snyder and Evans 2006; Tsutsui and Suarez 2003; Silverman and Brightwell 2008), but ant communities can also be subjected to invasions by other species of diverse taxa, such as plants (French and Major 2001; Marshall and Buckley 2009; Osunkoya et al. 2011). Information on the response of ants to invasive plants is inconsistent in the published data (Litt et al. 2014). On one hand, alien plants can enhance the establishment of some ant species if they increase seed or honeydew availability (Ostoja et al. 2009; Lescano and Farji-Brener 2011). On the other hand, as most studies demonstrated, the richness and abundance of ants tend to decrease in response to non-native plant invasions (Kutt and Fisher 2010; Litt et al. 2014). The proximate reasons for such decreases are poorly understood. Introduced plant species affect ant abundance, richness, and diversity indirectly by changes in the microhabitat, vegetation structure, and species interactions, as well as by altering prey availability (Gratton and Denno 2005; Kutt and Fisher 2010; Lenda et al. 2013; Mitrus et al. 2017).

*Myrmica rubra* is a eurytopic species, known from all Europe and Palaearctic Asia, and is one of the most common, native ant species in Poland (Czechowski et al. 2012). Today, the native habitats of *M. rubra*, especially wet meadows, are progressively being invaded by goldenrods, i.e., *Solidago canadensis, Solidago gigantea*, and *Solidago altissima*. The range of goldenrods in Europe increased threefold after the beginning of the spread that dates back to 1850 (Weber 1998). It was shown that ants develop smaller colonies and their foraging distance is longer in grasslands invaded by *Solidago* spp. compared to non-invaded meadows (Lenda et al. 2013). Similarly, the invasion has a profound effect on ant species richness and the structure of ant communities (Lenda et al. 2013; Kajzer-Bonk et al. 2016). Data presented by Kajzer-Bonk et al. (2016) showed that goldenrod invasion affects negatively mainly *Myrmica* ants, for which much lower number of nests were found in patches covered by these plant species.

An increasing body of evidence from diverse taxa suggests that invasions may drive evolutionary processes in both natives and intruders (Gilchrist and Lee 2007). Nevertheless, many studies focus on the biotic traits of the invaders, whereas little is known about the changes in life-history traits of native species in response to an invasion (Jones and Closs 2015). In the present study, we investigated wild colonies of the common ant *M. rubra* originating from grasslands invaded by goldenrods. The main aim was to assess if goldenrod invasion can affect colony size, total production, as well as other productivity parameters, i.e., growth and reproduction of *M. rubra* colonies. The production of workers, gynes, and males was compared between colonies living in grasslands invaded by goldenrods and colonies from semi-natural (non-invaded) grasslands.

## Materials and methods

### Study site

The study was carried out in the end of July/beginning of August 2013 and 2016 in an area located in a complex of wet grasslands of the ordo *Molinietalia caeruleae* near the city of Kraków (50°01′N/19°53′E). Recently, many meadows have been invaded by goldenrod as the effect of abandonment and the cessation of mowing (Moron et al. 2009). We selected 12 abandoned grasslands of two types: six invaded by goldenrod and six non-invaded. The selected sites were separated from each other by a distance of a minimum of 0.5 km to a maximum of 7 km. Invaded grasslands were dominated by goldenrod that covered between 90 and 100% of the soil surface (mix of *S. canadensis* L. and *S. gigantea* A.), whereas on non-invaded sites, only single plants of goldenrods were detected (less than 1% of surface).

### Myrmica ant colony size and productivity parameters

We excavated a total of 47 colonies of *Myrmica rubra* in the field, which then were taken into the laboratory: 23 colonies from non-invaded grasslands and 24 colonies collected from invaded grasslands. In 2016, before nest excavations, we also collected data on nest density. For each *M. rubra* colony found (*N* = 24: 12 invaded and 12 non-invaded), we assessed the density of all other ant nests in a square of 9 m^2^ with the chosen *M. rubra* nest in the middle. About 10 ants were taken in the laboratory for identification [using the key of Czechowski et al. (2012)] from each of the nests found in the squares. In the laboratory, for each colony, we counted: (1) the number of adult workers and queens; (2) the number of ant larvae; (3) the number of ant pupae, which were divided into worker pupae, male pupae, and queen (gyne) pupae; and (4) the number of winged queens and males. We did not categorize ant larvae into worker and sexual forms, but we assumed that in late July/beginning of August, most larvae belong to the worker caste (in *Myrmica* ants, sexual larvae develop in late spring and the beginning of summer; Elmes et al. 1998). This was also supported by the lack of large pre-pupae and the presence of well-colored male and gyne pupae, suggesting that the sexual forms pupated several days before the date of collection. Information on the total number of larvae, pupae, and winged sexual forms produced by the colony allowed us to calculate the colony production of particular brood types.

### Statistical analysis

Differences in the number of workers (colony size), adult queen number, and total production were analysed using generalized linear mixed models (GLMM) with a negative binominal error term. In the models, habitat type (invaded and non-invaded grasslands) was included as the fixed factor, whereas colony ID and year were included as random factors. The proportions of particular brood types to the total brood production of the ant colony were analysed by GLMM with binomial error, with habitat type as the fixed factor and colony ID and year as random factors. GLMMs were performed using the *glmer* function in the *lme4* package (Bates et al. 2013). We used the Pearson Chi-squared test with Yates correction to test the differences in the total number of new workers, gynes, and males produced by colonies from invaded and non-invaded grasslands.

To assess differences in nest density between invaded and non-invaded meadows, we used GLM models with negative binominal error term. In our models, the (a) number of all *Myrmica* nests and the (b) number of *M. rubra* nests around our colonies studied were included as dependent variables, whereas, in both models, the habitat type (invaded and non-invaded grasslands) was included as fixed factor.

All statistical analyses were performed in R Statistical Environment (R Core Team 2017).

## Results

**Fig. 1:**
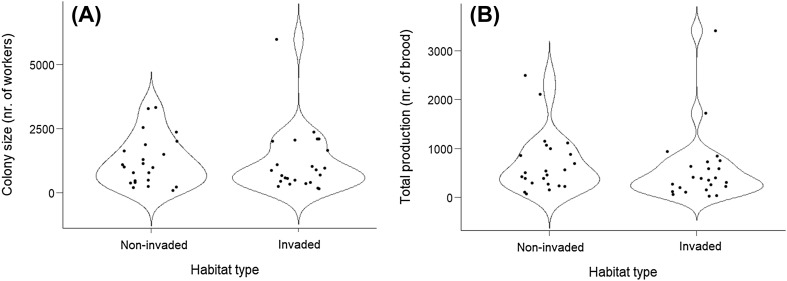
Violin plots with distribution of data points showing the number of workers—colony size (**a**) and total production (**b**) of *Myrmica rubra* colonies collected on grasslands invaded by goldenrod plants and non-invaded grasslands

*Myrmica rubra* colonies living in grasslands invaded by goldenrods were significantly smaller than colonies from non-invaded sites (GLMM *z* = − 2.06, *p* = 0.04, Table 1; Fig. 1a), but there was no difference in the number of adult queens (GLMM *z* = − 1.22, *p* = 0.23, Table 1). Total production was significantly lower in colonies from invaded grasslands than from non-invaded ones (GLMM *z* = − 2.63, *p* = 0.009, Table 1; Fig. 1b). All colonies were producing workers and the total number of new workers produced by colonies from non-invaded sites 14420, (mean ± SE 626.9 ± 116.06) were significantly higher than the number of new workers produced by colonies from invaded grasslands 10878, (mean ± SE 453.2 ± 135.44) (*χ*^2^ = 156, 2, *df* = 1, *p* < 0.001). The proportion of new workers to total brood production inside *M. rubra* colonies was not significantly different among colonies from the two habitat types (GLMM *z* = − 1.62, *p* = 0.104). In total, 1525 (mean ± SE 63.5 ± 20.95) gynes were produced by 50% of colonies from invaded grasslands, whereas 536 (mean ± SE 23.3 ± 12.64) gynes were produced by 30% of colonies from non-invaded sites. The total number of young queens produced by colonies from invaded grasslands was significantly higher compared to colonies from non-invaded grasslands (*χ*^2^ = 250. 8, *df* = 1, *p* < 0.001). In addition, the proportion of young queens to total brood production was significantly higher in colonies living in invaded sites compared to colonies from non-invaded grasslands (GLMM *z* = 1.935, *p* = 0.05). Males, 1076 in total, (mean ± SE 44.8 ± 19.25) were produced by 62% of colonies from invaded grasslands and by 65% of colonies from non-invaded sites (985 males in total, mean ± SE 42.8 ± 19.09) and these numbers were not significantly different (*χ*^2^ = 1.923, *df* = 1, *p* = 0.166). The proportion of males to total brood production also did not differ among colonies from invaded grasslands compared to colonies living in non-invaded ones (GLMM *z* = 0.471, *p* = 0.637).

**Table 1:** Summary of median, quartiles, and min–max values for colony size (number of workers), queen number, and productivity parameters of *Myrmica rubra* colonies coming from grasslands invaded by goldenrods (*N*_colony_ = 24) and non-invaded grasslands (*N*_colony_ = 23)

Parameters	Median and (first–third quartiles) and (min–max values) for colonies from non-invaded grasslands	Median and (first–third quartiles) and (min–max values) for colonies from invaded grasslands
Worker number	1012 (407–1878) (94–3327)	784 (426.5–1823.5) (152–5964)
Queen number	6 (3–13) (0–38)	4 (2–7.5) (1–43)
Total production	504 (268–994) (71–2494)	367 (176–680.5) (25–3416)
New worker production	418 (228–881) (71–2100)	274 (995–520.5) (25–3178)
Gyne production	0 (0–2) (0–248)	0.5 (0–118) (0–310)
Male production	3 (0–50) (0–393)	2 (0–60.5) (0–432)

The density of *Myrmica* nests around our studied colonies was significantly higher in non-invaded grasslands than in invaded ones (GLM *z* = − 2.26, *p* = 0.026). *M. rubra* nest density was also higher in non-invaded sites, but not on significant level (GLM, *z* = − 1.85, *p* = 0.06).

## Discussion

Our results showed that colonies living in invaded grasslands are smaller in size and consequently produce less brood in total than colonies from non-invaded sites. Moreover, it seems that colonies occurring in non-invaded grasslands invest more in maintenance, as total production of new workers is higher compared to colonies from invaded grasslands. The most interesting finding concerns the production of young queens, which is higher for colonies occurring in invaded sites. This result suggests that on the meta-population level, more new gynes are produced by colonies living in grasslands invaded by *Solidago* plants compared to colonies occurring in non-invaded sites. Moreover, in colonies from invaded grasslands, the proportion of gynes to all brood production is higher compared to colonies from non-invaded meadows. As male production seems to be at a similar level in all colonies, such results suggest that *M. rubra* colonies living in invaded grasslands switch their investment more into gyne production rather than into the production of young workers.

Our data were collected in the field, and thus, we are unable to assess the age of the colonies, but on the basis of the production parameters, we can assume that the age variation should be similar in both groups of colonies. All of them produced new workers, a similar proportion of colonies produced males, and some of the colonies produced gynes. According to life-history theory, an ant colony, analogically to single organisms, has to invest resources in maintenance, i.e., the production of workers, and in reproduction, i.e., the production of sexuals (Baroni-Urbani et al. 1978; Peakin and Josens 1978). In general, it is considered that the intensive production of workers occurs during the first stages of colony development, whereas sexual forms are produced later (Oster and Wilson 1978). One of the reasons for this is that the production of sexuals is costly in terms of feeding and the foraging effort of workers. The production of gyne pupa requires more resources compared to worker or male pupa (Boomsma and Isaaks 1985; Grześ and Okrutniak 2016). It is also known that the production of sexual forms in ant colonies can be correlated with various factors, such as colony size, queen age, food availability, and habitat conditions (Nielsen and Josens 1978). Because our findings are based on field studies, the reason underlying this difference can be attributed to various environmental factors, but it can also be discussed in terms of the adaptive colony-level response of ants to stress and all possible interpretations should be evaluated with caution.

The previous studies highlighted that goldenrod-invaded grasslands are not suitable for *Myrmica* ants or can even be considered stressful (Lenda et al. 2013; Kajzer-Bonk et al. 2016). According to these studies, the density of *Myrmica* nests was lower, estimated colony size was smaller and the foraging distance of ants was longer in such grasslands compared to non-invaded sites. However, certain life-history traits of ants emphasize their plasticity in the face of environmental disturbance and, therefore, may have adaptive significance. Many such traits may operate at the colony level. Walin et al. (2001) reported that colonies of *M. rubra* may respond to their spatial position and nest density. Colonies of high nest density maintained more queens per nest, and thus, site limitation can promote polygyny. In general, possible traits that help to overcome frequent disturbances include small colony size, fast generation time, polygyny, polydomy, and morphologically unspecialized workers, but there are many notable exceptions to this generalization (Linksvayer and Janssen 2009). It also seems that maintaining morphologically unspecialized workers is more efficient for dealing with habitat disturbances (Hölldobler and Wilson 2009; Bourke and Franks 1995). *Myrmica rubra* shares all the above traits, suggesting the high robustness of this species. It is also likely that the higher production of gynes observed in invaded grasslands supports dispersal and, therefore, might be a colony-level mechanism of avoiding the disturbed habitat and enhancing the probability of establishing a colony in a more favourable location. Therefore, the observed pattern of gyne production may be a result of the species’ plasticity, favouring its performance in the disturbed environment of goldenrod invasion.

Lower gyne production in colonies from non-invaded grasslands compared to those from invaded sites may also be due to the effect of local resource competition among related queens in non-invaded grasslands. As showed our data, *Myrmica* nest density is higher in non-invaded grasslands, which in turn may lead to higher competition among inseminated gynes for a nest site compared to invaded grasslands. It is known that this phenomenon often occurs in polygynous ant species, like, e.g., *M. rubra*. In addition, it is known that *Myrmica* colonies are commonly established by the fragmentation of an existing colony (Elmes et al. 1998), when one queen leaves the nest with part of the workers. In such cases, the worker force becomes part of the investment in females (Craig 1980; Bulmer 1983), which in turn may also partly explain the greater number of new workers produced by colonies living in the control grasslands. It is interesting to note that the density of *M. rubra* nests in non-invaded sites is also higher (although not on significant level of 0.05) than in invaded grasslands. This fact may suggest that *Solidago* invasion can also change *M. rubra* population structure; as in non-invaded places, most of colonies can be polydomous and create supercolonial units, whereas in invaded sites, *Myrmica* colonies seem to be more separated from each other. This in turn may also influence observed changes in the reproductive strategies of ant colonies.

To conclude, our study suggests that the invasion of goldenrod plants can be an important factor influencing the reproductive strategies of *Myrmica* ant colonies. It seems that in such changed habitats, ants may invest more in reproduction by producing more gynes and promote dispersal. As a trade-off, this higher investment into actual reproduction can lead to a situation where colonies may invest less energy in colony growth, produce fewer workers, and attain smaller colony size.
